# TRPA1 Promotes Cardiac Myofibroblast Transdifferentiation after Myocardial Infarction Injury via the Calcineurin-NFAT-DYRK1A Signaling Pathway

**DOI:** 10.1155/2019/6408352

**Published:** 2019-05-14

**Authors:** Shuang Li, Xiongshan Sun, Hao Wu, Peng Yu, Xin Wang, Zhenhua Jiang, Erhe Gao, Jiangwei Chen, De Li, Chenming Qiu, Baomei Song, Ken Chen, Kecheng He, Dachun Yang, Yongjian Yang

**Affiliations:** ^1^Department of Cardiology, The General Hospital of Western Theater Command (Chengdu Military General Hospital), Chengdu 610083, China; ^2^Department of Toxicology, The Ministry of Education Key Laboratory of Hazard Assessment and Control in Special Operational Environment, Shaanxi Key Laboratory of Free Radical Biology and Medicine, School of Public Health, Fourth Military Medical University, Xi'an, Shaanxi 710032, China; ^3^Department of Cardiology, Xijing Hospital, Fourth Military Medical University, Xi'an, Shaanxi 710032, China; ^4^Department of Anesthesiology and Perioperative Medicine, Xijing Hospital, Fourth Military Medical University, Xi'an, Shaanxi 710032, China; ^5^Center of Translational Medicine, Temple University School of Medicine, Philadelphia, PA 19107, USA

## Abstract

Cardiac fibroblasts (CFs) are a critical cell population responsible for myocardial extracellular matrix homeostasis. After stimulation by myocardial infarction (MI), CFs transdifferentiate into cardiac myofibroblasts (CMFs) and play a fundamental role in the fibrotic healing response. Transient receptor potential ankyrin 1 (TRPA1) channels are cationic ion channels with a high fractional Ca^2+^ current, and they are known to influence cardiac function after MI injury; however, the molecular mechanisms regulating CMF transdifferentiation remain poorly understood. TRPA1 knockout mice, their wild-type littermates, and mice pretreated with the TRPA1 agonist cinnamaldehyde (CA) were subjected to MI injury and monitored for survival, cardiac function, and fibrotic remodeling. TRPA1 can drive myofibroblast transdifferentiation initiated 1 week after MI injury. In addition, we explored the underlying mechanisms via *in vitro* experiments through gene transfection alone or in combination with inhibitor treatment. TRPA1 overexpression fully activated CMF transformation, while CFs lacking TRPA1 were refractory to transforming growth factor *β*- (TGF-*β*-) induced transdifferentiation. TGF-*β* enhanced TRPA1 expression, which promoted the Ca^2+^-responsive activation of calcineurin (CaN). Moreover, dual-specificity tyrosine-regulated kinase-1a (DYRK1A) regulated CaN-mediated NFAT nuclear translocation and TRPA1-dependent transdifferentiation. These findings suggest a potential therapeutic role for TRPA1 in the regulation of CMF transdifferentiation in response to MI injury and indicate a comprehensive pathway driving CMF formation in conjunction with TGF-*β*, Ca^2+^ influx, CaN, NFATc3, and DYRK1A.

## 1. Introduction

Cardiovascular diseases remain the leading cause of mortality worldwide, with myocardial infarction- (MI-) based injury and subsequent left ventricular (LV) remodeling and heart failure as the major sequelae underlying this lethality [1]. After MI, physiological compensatory mechanisms promote cardiomyocyte loss and fibrosis via pathological remodeling [2]. Cardiac fibroblasts (CFs) are a key cellular component of post-MI LV remodeling. In response to cardiac injury or stress, CFs undergo directly programmed conversion into cardiac myofibroblasts (CMFs), a process associated with increased secretion of extracellular matrix (ECM) components and critical to maintain ventricular wall structural integrity and to reduce dilation at the early stage of postinfarction remodeling [3]. CMFs likely become contractile as a result of newly acquired expression of genes such as smooth muscle *α*-actin (*α*-SMA) [4]. Periostin is another described marker of CMFs that is expressed in adult heart tissues only after injury [5].

CMFs arise from the transdifferentiation of CFs because injured heart tissues contain a variety of mechanical and cytokine/neurohumoral signals; one of fundamental initiators is the cytokine transforming growth factor *β* (TGF-*β*) [6]. TGF-*β* initiates intracellular signaling through both canonical and noncanonical signaling pathways [7]. Recently, another important activator of myofibroblast differentiation was identified [8]. Transient receptor potential (TRP) channels comprise a superfamily of cation channels (TRPC (canonical), TRPM (melastatin), TRPV (vanilloid), TRPP (polycystin), TRPA (ankyrin), and TRPML (mucolipin)) [9]. The expression of TRP channels in CFs has been reported, but the functional role of TRP channels and their contribution to the pathogenesis of cardiac remodeling is poorly understood [10].

TRPA1, the sole member of the mammalian ankyrin TRP subfamily, is a large-conductance, Ca^2+^-permeable, nonselective cation channel [11]. TRPA1 is widely expressed in several neural tissues and is considered a key player in (neuropathic) pain, inflammation, and the response to cold [12]. In genome-wide association studies, TRPA1 displays a suggestive association with coronary artery disease [13]. Recently, TRPA1 was reported to be implicated in cardiac fibrosis [14]. In primary human ventricular cardiac fibroblasts, methylglyoxal provokes a sustained increase in the intracellular Ca^2+^ concentration that is greatly reduced by treatment with HC030031, a selective TRPA1 antagonist, or by siRNA-induced knockdown of TRPA1 [14]. Additionally, TRPA1-selective inhibitors protected against cardiac hypertrophy and fibrosis by modulating M2 macrophage differentiation [15]. However, the potential effects and mechanisms of TRPA1 in cardiac fibrosis after MI have not been explored. Clearly, a deeper understanding of the responsible signaling pathways is necessary to derive improved treatment options. Here, we demonstrate that TRPA1 is an inducing factor of CF transdifferentiation into CMF during MI injury. TRPA1 deletion blocks myofibroblast formation *in vivo* and *in vitro* in response to MI injury and TGF-*β* stimulation.

## 2. Materials and Methods

### 2.1. Animal Modeling and Grouping

Male Trpa1 knockout (*Trpa1^−/−^*) (KO, stock number 008646) mice (8-10 weeks old) were obtained from Jackson Laboratory (Bar Harbor, Maine, USA). Male wild-type littermate (*Trpa1^+/+^*) (WT) mice (8-10 weeks old) and KO mice were screened and housed under a 12 h : 12 h light-dark cycle at 22°C with free access to food and water. All experiments were performed in adherence with the National Institutes of Health Guidelines on the Care and Use of Laboratory Animals and were approved by the Fourth Military Medical University Committee on Animal Care (ID: 2013052).

Cinnamaldehyde (CA) (Sigma-Aldrich, USA) was dissolved in saline containing 0.2% dimethyl sulfoxide (DMSO). KO and WT mice were randomly allocated into the following groups with *n* = 25 mice each: (1) the WT+sham group (WT Sham); (2) the KO+sham group (KO Sham); (3) the WT+sham+CA group (CA Sham); (4) the WT+MI group (WT MI); (5) the KO+MI group (KO MI); and (6) the WT+MI+CA group (CA MI). The CA Sham group and CA MI group were intraperitoneally (i.p.) injected daily with CA at a dose of 50 mg/kg body weight for 4 weeks before surgery [16]. The mice in the sham groups were injected with saline at the same volume instead.

The mouse model of MI was induced by ligation of the left anterior descending (LAD) artery [17]. In brief, mice were anesthetized via continuous inhalation of 2% isoflurane during the operation. A left thoracotomy was performed, and the pericardium was opened. The LAD was permanently ligated with a 6-0 suture at the level of the left atrium. The ligation was deemed successful when the anterior wall of the LV turned pale. Characteristic echocardiographic changes were utilized to further confirm the establishment of the mouse MI model. Sham group mice underwent the same surgical procedures without the LAD suture.

### 2.2. Cardiac Function Evaluation by Echocardiography

Echocardiography was used to assess ventricular function 1 week after MI surgery. Echocardiography was performed under anesthesia using a 30 MHz transducer on a Vevo 2100 ultrasound system (VisualSonics, Canada) as previously described [18].

### 2.3. Evaluation of Fibrosis

Mice were euthanized, and hearts were harvested for histological staining at 7 days after surgery. Masson's trichrome staining and Sirius red staining were used to assess cardiac fibrosis as previously described [19, 20].

### 2.4. Determination of TGF-*β* Levels

TGF-*β* levels in cardiac LV tissue were spectrophotometrically measured with commercial ELISA assay kits (R&D Systems, USA) according to the manufacturer's instructions.

### 2.5. Cardiac Fibroblast Isolation, Culture, Treatment, and Transfection

Primary cultures of CFs were obtained from 1- to 2-day-old neonatal or adult *Trpa1^−/−^* and *Trpa1^+/+^* mice by enzymatic digestion of the left ventricle. The isolation, culture, and purity assessment of CFs were performed as previously described [21]. CFs were seeded in plates or dishes at 70% confluency and serum-starved overnight with media containing 1% FBS; CMF transformation was then induced using 10 ng/mL recombinant porcine TGF-*β* (R&D Systems, USA) for 24-48 hr [22]. For overexpression of the *Trpa1* gene, CFs were incubated with adenovirus (Shanghai GeneChem Co. Ltd., China). CFs were maintained in complete medium, and antibiotics were removed before adenovirus infection. Cells were transfected with Ad-TRPA1 or Ad-NC virus at a multiplicity of infection (MOI) of 100. Cells not transfected with virus were classified as the control group. 24 hr after infection, the medium was replaced with complete medium for an additional 48 hr before cells were harvested. Successful infection was ascertained by determining the expression of TRPA1 by Western blotting. Knockdown of *Trpa1* expression in CFs was achieved by siRNA targeting mouse CFs (GenePharma, China). A scrambled RNA sequence was used as the control. Transfection was performed according to the manufacturer's instructions. For experiments involving inhibitors, the calcineurin (CaN) inhibitor FK506 (InvivoGen, USA) (10 *μ*M) or the DYRK1A inhibitor harmine (Sigma-Aldrich, USA) (10 *μ*M) was added to the medium 3 h before TGF-*β* treatment to ensure maximum inhibition [23].

### 2.6. Immunofluorescence Staining

CMF transdifferentiation was scored by *α*-SMA (1 : 100, rabbit monoclonal antibody, Abcam, USA) and CD 31 (1 : 100, rabbit polyclonal antibody, Abcam) stress fiber formation via immunofluorescence staining as described previously [19].

### 2.7. TRPA1 Luciferase Reporter and Luciferase Activity Assay

CFs (10^5^ cells/mL) were plated into 6-well plates. Cells were transiently cotransfected with plasmid containing the mouse *α*-SMA promoter linked to firefly luciferase (Shanghai GeneChem Co. Ltd., China) using TurboFect Transfection Reagents (Thermo Fisher Scientific, USA) according to the manufacturer's protocol. Briefly, serum-free DMEM containing *α*-SMA promoter-luciferase plasmids or empty plasmids was mixed with TurboFect transfection reagents and added to the CFs. After 8 hr, cells were incubated with DMEM containing 10% FBS. 48 hr after transfection, cells were lysed with Passive Lysis Buffer (Promega, USA), and transcriptional activity was measured using the luciferase assay system (Promega, USA) with a GloMax 20/20 luminometer (Promega, USA).

### 2.8. Collagen Gel Contraction Assay

CF contractile activity was assessed by a CytoSelect™ Cell Contraction Assay Kit (Cell Biolabs Inc., USA). CFs were harvested and resuspended in medium at 2-5 × 106 cells/mL. The cell contraction matrix was prepared by mixing 2 parts of cell suspension with 8 parts cold Collagen Gel Working Solution. Then, 250 *μ*L of the cell contraction matrix was added to each well of the 48-well plate. The plate was transferred to 37°C and 5% CO_2_ for 1 hr. After collagen polymerization, medium (with/without contraction mediators) was placed atop each collagen gel lattice. Wells were monitored for contraction for 2 days at 37°C and 5% CO_2_. Medium was changed daily by carefully removing 250 *μ*L and replacing with 250 *μ*L of fresh medium (with/without contraction mediators). The change in the size of the collagen gel was measured and quantified with ImageJ software (NIH, USA).

### 2.9. Measurement of the Cytosolic Ca^2+^ Concentration

A Fluo-4 NW Calcium Assay Kit (Invitrogen, USA) was used to monitor the cytosolic Ca^2+^ concentration in CFs according to the manufacturer's instructions. Ca^2+^ imaging was performed on a confocal laser scanning microscope (Olympus FluoView™ FV1000, Japan). Images were acquired at 1 frame/second. The *F*_0_ value was determined by averaging the fluorescence values from 10 consecutive baseline images. Images were analyzed and quantitated using the Olympus FluoView software.

### 2.10. Cellular CaN Activity Assay

CFs were treated and dissociated. Cell extracts were prepared using reagents provided in the CaN cellular activity assay kit (Enzo Life Sciences Inc., USA). The assay was performed following the manufacturer's instructions. The absorbance values from the assay were converted into the amount of released phosphate according to the manufacturer's instructions.

### 2.11. Western Blot

Total protein was extracted from cardiac ventricular tissues or freshly isolated/cultured CFs using RIPA lysis buffer (TIANGEN, China). Cytoplasmic and nuclear protein fractions were extracted from CFs with a Nuclear Extraction Assay Kit (Thermo Fisher Scientific, USA). Briefly, CFs were collected, washed, and transferred into a prechilled microcentrifuge tube. Cells were gently resuspended in 500 *μ*L of 1x hypotonic buffer by pipetting and were incubated on ice for 15 minutes. A 25 *μ*L volume of detergent (10% NP40) was added, and the mixture was vortexed for 10 seconds at the highest setting. The homogenate was centrifuged for 10 minutes at 3,000 rpm and 4°C. The supernatant was transferred and saved. This supernatant contained the cytoplasmic fraction. The pellet contained the nuclear fraction. The nuclear pellet was resuspended in 50 *μ*L of complete Cell Extraction Buffer for 30 minutes on ice with vortexing at 10-minute intervals and was then centrifuged for 30 minutes at 14,000 ×g and 4°C. The supernatant (nuclear fraction) was transferred to a clean microcentrifuge tube. The protein concentration was determined using the BCA method (Thermo Fisher Scientific, USA). Western blotting was performed following a standard protocol, as described previously [24]. The following primary antibodies were used: anti-TRPA1 (110 kDa, 1 : 1000, rabbit polyclonal, Novus Biologicals, USA), anti-Postn (93 kDa, 1 : 1000, rabbit polyclonal, Novus Biologicals, USA), anti-NFATc3 (115 kDa, 1 : 1000, rabbit polyclonal, Abcam, USA), anti-DYRK1A (86 kDa, 1 : 1000, rabbit polyclonal, Abcam, USA), anti-*β*-actin (43 kDa, 1 : 1,000, rabbit polyclonal, Santa Cruz Biotechnology Inc., USA), anti-GAPDH (36 kDa, 1 : 1000, rabbit monoclonal, Abcam, USA), and anti-Histone H3 (15 kDa, 1 : 1000, rabbit monoclonal, Abcam, USA). A horseradish peroxidase-conjugated goat anti-rabbit secondary antibody (Zhongshan Biotechnology Co. Ltd., China) was used.

### 2.12. Statistical Analysis

Data are expressed as the means ± SDs and were analyzed using ANOVA followed by a post hoc *t*-test with Bonferroni correction. The survival rate was analyzed via the Kaplan-Meier method followed by the log-rank post hoc test. All statistical tests were performed using SPSS software version 17.0 (IBM, Armonk, USA) and GraphPad Prism software version 6.0 (GraphPad Software, CA). A value of *P* < 0.05 was considered statistically significant.

## 3. Results

### 3.1. TRPA1 Is Necessary for MI Injury-Induced Cardiac Fibrosis

**Figure 1:**
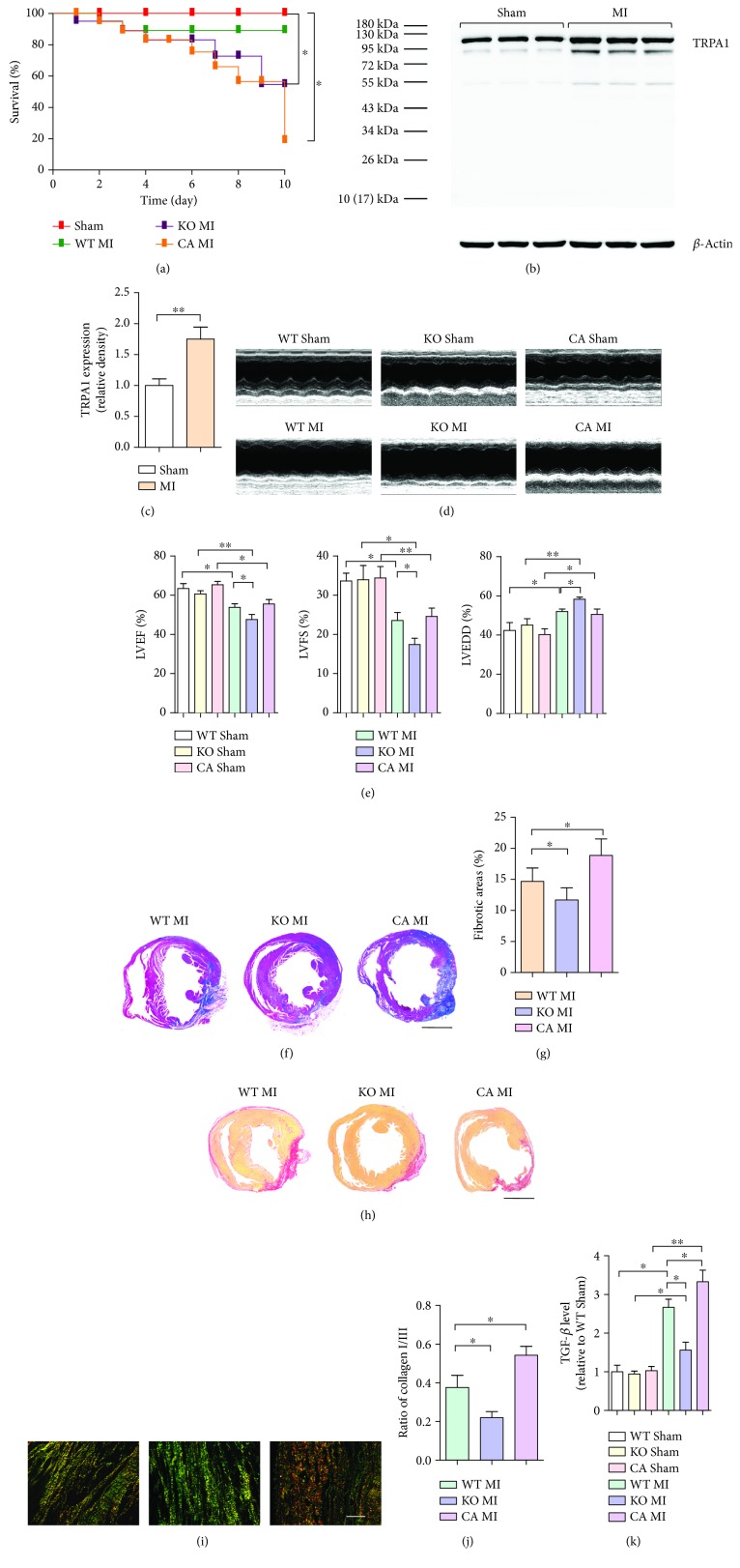
Effect of TRPA1 on the course of cardiac fibrosis after MI injury. (a) Survival curves of *Trpa1^+/+^* (WT), Trpa1^−/−^ (KO), and CA-pretreated WT (CA) littermates after sham or permanent coronary ligation surgery (MI). *n* = 20 per group. (b and c) Western blot analysis and quantification of the TRPA1 protein level in the ventricles of MI-injured mice. Molecular weights in kDa are shown to the right of the blots. *n* ≥ 3 per group. (d) Representative M-mode echocardiography images of mice. (e) Measurement of the left ventricular ejection fraction (LVEF), left ventricular fractional shortening (LVFS), and left ventricular end-diastolic dimension (LVEDD). *n* ≥ 10 per group. (f) Representative images of Masson's trichrome staining. Red, viable myocardium; blue, fibrosis due to infarction damage (scale bar = 1 mm). (g) Quantitative analysis of the fibrotic area (blue). *n* ≥ 5 per group. (h) Picrosirius red staining for collagens in sections of myocardium as observed via optical microscopy (scale bar = 1 mm). (i) Under polarized light, the orange and red colors indicate type I collagen deposition and the green and yellow colors indicate type III collagen deposition in the myocardial infarct region (scale bar = 25 *μ*m). (j) Collagen subtype ratio in the infarct region as calculated by image analysis. *n* ≥ 5 per group. (k) The TGF-*β* level in the myocardial tissue lysis supernatant was quantified by ELISA. *n* ≥ 4 per group. The error bars represent the means ± s.e.m.^∗^ *P* < 0.05 and ^∗∗^ *P* < 0.01. The data are representative of the results of three or more independent experiments.

3 to 7 days after MI injury is considered to be the time during which scar formation and remodeling mostly occur. To investigate the potential role of TRPA1 in cardiac fibrosis induced by MI injury, we first examined the expression of TRPA1 in mouse ventricular tissue on the 7th day after MI injury. Our results showed that TRPA1 expression dramatically increased in MI mouse ventricular tissue compared with that in uninjured control mouse tissue (*P* < 0.05) (Figures 1(b) and 1(c)). *Trpa1^−/−^* mice (KO MI) and CA-pretreated mice (CA MI) had a higher mortality rate than *Trpa^+/+^* mice (WT MI) subjected to MI injury (*P* < 0.05 and *P* < 0.05, respectively) (Figure 1(a)). The surviving KO MI mice had greater reductions in cardiac function and observably greater ventricular wall dilation than the WT MI mice, as measured by echocardiography (*P* < 0.05, *P* < 0.05, and *P* < 0.05) (Figures 1(d) and 1(e)). However, compared to WT MI mice, KO MI mice had significant reductions in fibrotic scarring, as determined by Masson's trichrome staining (*P* < 0.05, *P* < 0.01, and *P* < 0.05) (Figures 1(f) and 1(g)); collagen content, as reflected by picrosirius red staining (Figures 1(h), 1(i), and 1(j)); and TGF-*β* levels in heart tissue lysate supernatant (Figure 1(k)) (*P* < 0.05, *P* < 0.05, and *P* < 0.01, respectively), whereas CA pretreatment aggravated cardiac fibrosis (Figures 1(f) and 1(j)), indicating that TRPA1 is necessary for MI injury-induced cardiac fibrosis.

### 3.2. TRPA1 Plays an Important Role in CMF Transformation and Cardiac Fibrosis Post-MI

**Figure 2:**
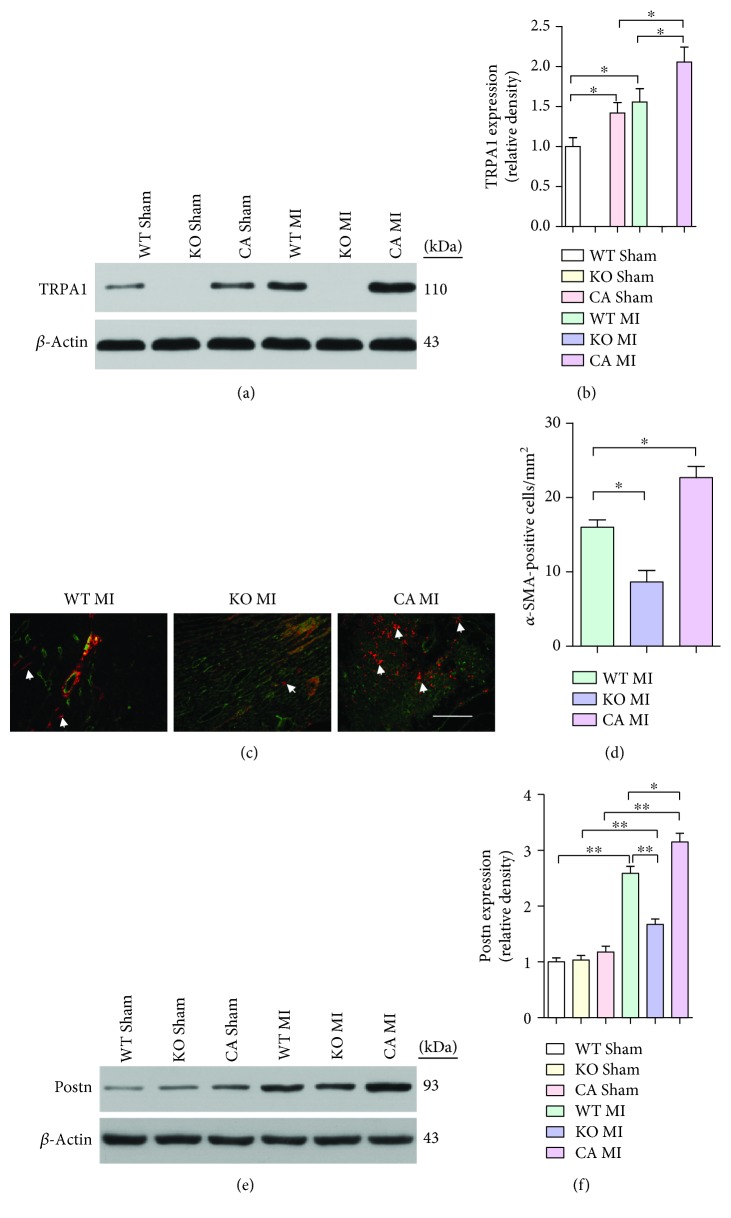
TRPA1-mediated CMF activation in the fibrotic response to MI injury. (a and b) Western blot analysis and quantification of TRPA1 protein expression from adult CFs isolated from the ventricles of sham or MI-injured mice. Molecular weights in kDa are shown to the right of the blots. *n* ≥ 3 per group. (c and d) Immunofluorescence staining and quantification of CMF numbers (white arrows) in the cardiac infarction border zone 7 days after injury. Myofibroblasts are positive for *α*-SMA (red) and negative for CD-31 (green) (scale bar = 25 *μ*m). Five sections were measured per heart, *n* ≥ 3 per group. (e and f) Western blot analysis and quantification of Postn protein expression in adult CFs isolated from the ventricles of sham or MI-injured mice. Molecular weights in kDa are shown to the right of the blots. *n* ≥ 3 per group. The error bars represent the means ± s.e.m.^∗^ *P* < 0.05 and ^∗∗^ *P* < 0.01. The data are representative of the results of three or more independent experiments.

MI injury induced TRPA1 expression in CFs isolated from the ventricle of mice in the MI groups relative to that in sham mice (*P* < 0.05) (Figures 2(a) and 2(b)). The border zone was analyzed for the presence of CMFs using immunofluorescent imaging of *α*-SMA (red) and CD31, a marker of endothelial cells (green), which showed that the KO MI group had fewer CMFs than the WT MI group (*P* < 0.05) (Figures 2(c) and 2(d)). Periostin (Postn) has been used to mark essentially all newly activated fibroblasts (myofibroblasts) but not unactivated fibroblasts within the heart during an injury event [25]. Postn expression in CFs isolated from mouse ventricles was decreased in the KO MI group compared with that in the WT MI group (*P* < 0.01) (Figures 1(e)and 2(f)). CA pretreatment aggravated CMF formation (Figures 2(c) and 2(f)). These results suggest that TRPA1-mediated transdifferentiation is vital to CFs through inducing CMF formation.

### 3.3. Loss of TRPA1 Prevents Consistent TGF-*β*-Mediated CMF Transdifferentiation

**Figure 3:**
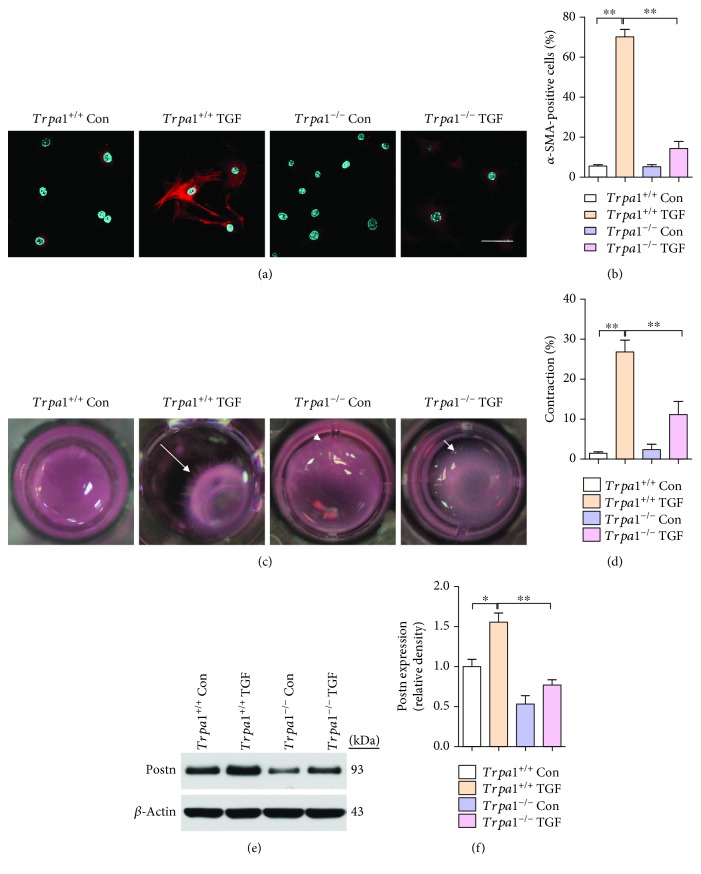
Deletion of TRPA1 in resident CFs abrogates TGF-*β*-mediated CMF transdifferentiation. (a and b) Immunofluorescence staining (a) and quantification (b) of *α*-SMA-positive (red) stress fibers and DAPI (blue) in neonatal *Trpa1^+/+^* and *Trpa1^−/−^* mouse primary CFs with and without TGF-*β* stimulation (scale bars = 25 *μ*m). (c and d) Images (c) and quantification (d) of the contraction of floating collagen gel matrices seeded with *Trpa1^+/+^* or *Trpa1* ^−/−^ CFs at 24 hr after TGF-*β* stimulation. The white arrows show the direction of contraction. *n* ≥ 3 per group. (e and f) Western blot analysis and quantification of Postn protein expression in primary *Trpa1^+/+^* and *Trpa1^−/−^* CFs, with and without TGF-*β* stimulation. Molecular weights in kDa are shown to the right of the blots. *n* ≥ 3 per group. The error bars represent the means ± s.e.m.^∗^ *P* < 0.05 and ^∗∗^ *P* < 0.01. The data are representative of the results of three or more independent experiments.

To determine whether TRPA1 is required for myofibroblast conversion, CFs isolated from neonatal Trpa1^−/−^ and *Trpa1^+/+^* littermates were examined for TGF-*β*-induced *α*-SMA stress fiber formation, Postn expression, and contractile function. Notably, *Trpa1^−/−^* primary CFs showed no induction of *α*-SMA-positive stress fiber formation with TGF-*β* treatment, in contrast to the observable induction in similarly prepared *Trpa1^+/+^* CFs (*P* < 0.01) (Figures 3(a) and 3(b)). The function of CMFs is to contract a collagen gel matrix; TGF-*β* induced a profound contraction of collagen gels cultured with *Trpa1^+/+^* CFs, while collagen gels cultured with *Trpa1^−/−^* CFs were refractory to TGF-*β*-mediated contraction (*P* < 0.01) (Figures 3(c) and 3(d)). In addition, Postn expression was decreased in the *Trpa1^−/−^* group compared with that in the *Trpa1^+/+^* group (*P* < 0.01) (Figures 3(e) and 3(f)). Moreover, TGF-*β* treatment causes a small increase in *α*-SMA-positive cells in *Trpa1* ^−/−^ CFs, as well as an increase in collagen contraction when compared to control *Trpa1* ^−/−^ CFs. However, there was no significant difference between these two groups (Figures 3(a) and 3(d)). This suggests that at least part of the effects of TGF-*β* are independent of TRPA1 channels but may be mediated by other Ca^2+^-permeable TRP channels [8].

### 3.4. TRPA1 Overexpression Promotes CMF Transdifferentiation

**Figure 4 fig4:**
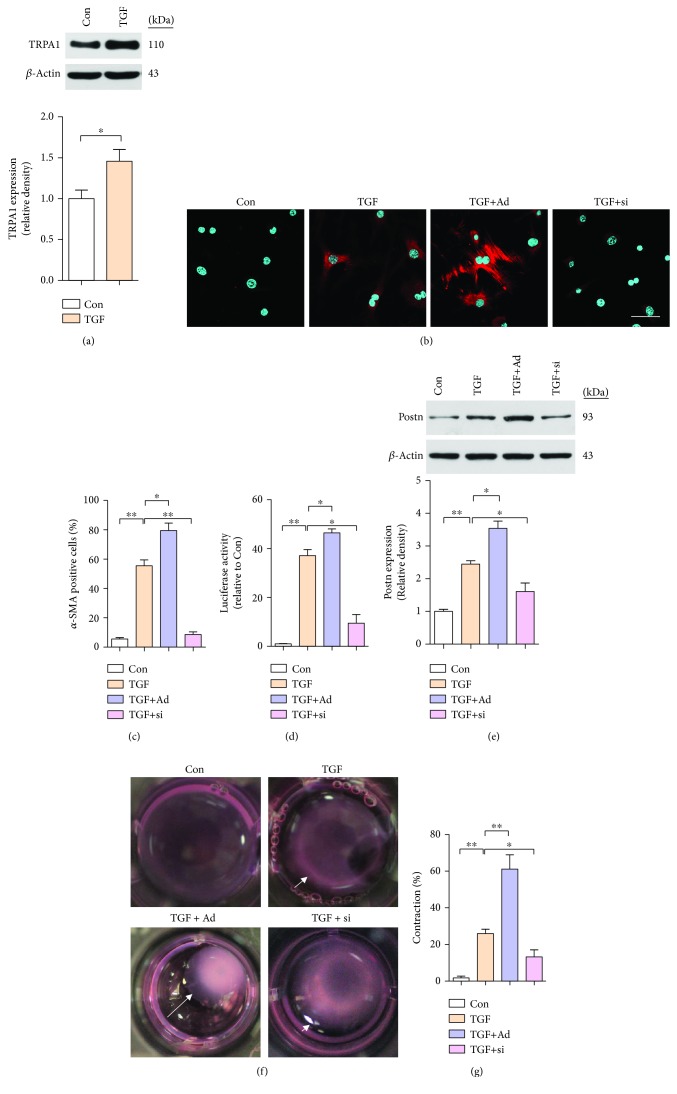
Overexpression of TRPA1 in primary CFs enhances CMF transdifferentiation. (a) Western blot analysis and quantification of TRPA1 protein expression in neonatal WT mouse primary CFs with and without TGF-*β* stimulation. Molecular weights in kDa are shown to the right of the blots. *n* ≥ 3 per group. (b and c) Immunofluorescence staining (b) and quantification (c) of *α*-SMA-positive (red) stress fibers and DAPI (blue) in CFs treated with TGF-*β* transfected with negative control (NC) adenoviral vector (not shown), Ad-TRPA1 (Ad), negative control (NC) siRNA (not shown), or si-TRPA1 (si) and treated 48 hr later with TGF-*β*. (scale bars = 25 *μ*m). *n* ≥ 3 per group. (d) *α*-SMA-luciferase promoter activity in CFs treated as described above. *n* ≥ 3 per group. (e) Western blot analysis and quantification of Postn protein expression in CFs treated as described above. Molecular weights in kDa are shown to the right of the blots. *n* ≥ 3 per group. (f and g) Images (f) and quantification (g) of the contraction of floating collagen gel matrices seeded with CFs treated as described above. *n* ≥ 3 per group. The error bars represent the means ± s.e.m.^∗^ *P* < 0.05 and ^∗∗^ *P* < 0.01. The data are representative of the results of three or more independent experiments.

To examine the requirement for TRPA1 in mediating TGF-*β*-dependent myofibroblast transdifferentiation, we used recombinant adenoviral TRPA1 vector (Ad-TRPA1, Ad) or siRNA (si-TRPA1, si) to overexpress or knock down TRPA1, respectively (Figure S1). Western blot analysis showed that TGF-*β* stimulation enhanced the protein levels of TRPA1 in primary CFs from neonatal WT mice (*P* < 0.05) (Figure 4(a)). To investigate whether TRPA1 can directly induce the transdifferentiation of CFs into CMFs, we utilized an *α*-SMA-luciferase promoter plasmid as a surrogate for CMF activation. We infected CFs with Ad-TRPA1 and si-TRPA1, treated them 48 hr later with TGF-*β*, and then assessed *α*-SMA stress fiber formation, Postn expression, and contractile function. TRPA1 overexpression induced *α*-SMA stress fiber positivity and CMF conversion (*P* < 0.05 and *P* < 0.05, respectively) (Figures 4(b) and 4(d)). In addition, compared with TGF-*β* treatment only, Ad-TRPA1 transfection enhanced the expression of Postn, as determined by Western blotting (*P* < 0.05) (Figure 4(e)). However, si-TRPA1 transfection attenuated the effect of CMF formation (Figures 4(b) and 4(e)). Moreover, Ad-TRPA1-transfected CFs showed collagen gel matrix contraction, while siRNA-transfected CFs did not (*P* < 0.01 and *P* < 0.05) (Figures 4(f) and 4(g)).

### 3.5. TRPA1 Overexpression Induces Cytosolic Calcium Signaling in CFs

**Figure 5 fig5:**
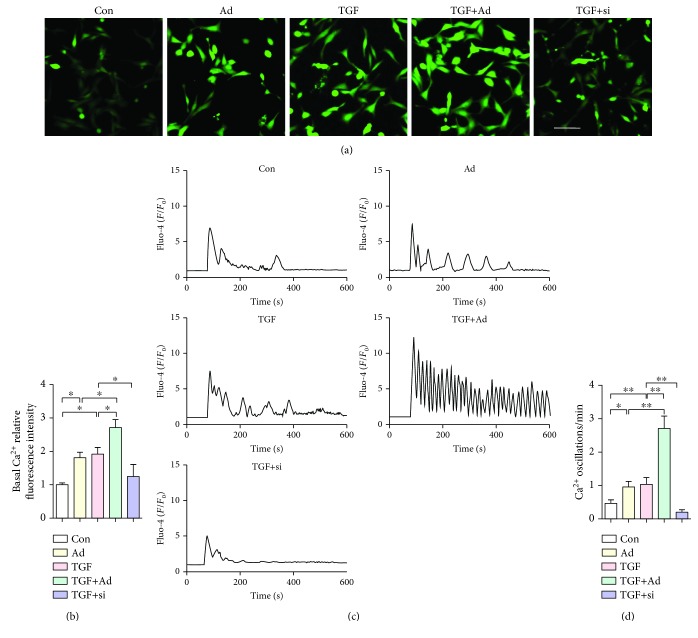
TRPA1 overexpression stimulates Ca^2+^ influx events in CFs. (a and b) Immunofluorescence staining (a) and quantification (b) of Fluo-4 in neonatal WT mouse primary CFs treated with or without TGF-*β* or transfected with Ad-TRPA1 (Ad) or si-TRPA1 (si) and treated 48 hr later with TGF-*β* (scale bars = 25 *μ*m). *n* ≥ 3 per group. (c and d) Representative traces (c) and frequencies (d) of Ca^2+^ oscillation (*n* = 50 cells) in single-cell Ca^2+^ imaging experiments. Ca^2+^ signals are presented as *F*/*F*_0_ values, where *F* is the Fluo-4 fluorescence value after treatment and *F*_0_ is the value before treatment. *n* ≥ 3 per group. The error bars represent the means ± s.e.m.^∗^ *P* < 0.05 and ^∗∗^ *P* < 0.01. The data are representative of the results of three or more independent experiments.

To investigate the role of TRPA1 in cytosolic calcium signaling, as mentioned earlier, CFs were transfected with Ad-TRPA1 or siRNA for 48 hr and were then treated with TGF-*β*. Furthermore, we measured the cytosolic Ca^2+^ concentration ([Ca^2+^]c) and Ca^2+^ oscillation by Fluo-4 staining. Fluorescence signals representing the [Ca^2+^]c showed significant increases with Ad-TRPA1 transfection and TGF-*β* treatment compared to their levels in cells treated with only TGF-*β* or only Ad-TRPA1 transfection (*P* < 0.05 and *P* < 0.05) (Figures 5(a) and 5(b)). Representative traces and quantification showed that TRPA1 overexpression significantly promoted TGF-*β*-dependent Ca^2+^ oscillation (*P* < 0.01) (Figures 5(c) and 5(d)). Moreover, this TGF-*β*-dependent increase in the cytosolic Ca^2+^ signal in CFs was reduced with si-TRPA1 transfection (Figures 5(a) and 5(d)). Our data indicated that TRPA1 is the primary mediator of the TGF-*β*-dependent enhancement of the cytosolic calcium signal in activated CFs.

### 3.6. TRPA1 Regulates the Ca^2+^-CaN-NFAT Pathway for CMF Transdifferentiation

**Figure 6 fig6:**
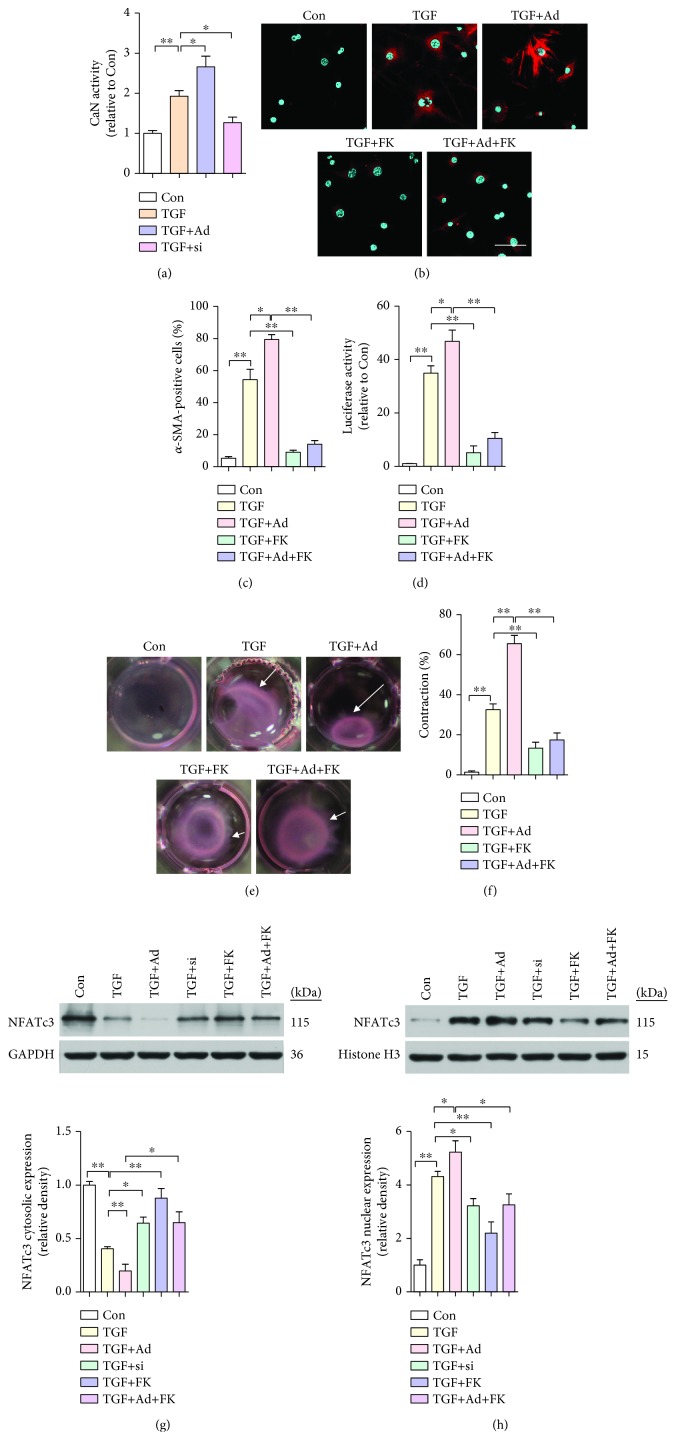
TRPA1 mediates CMF transdifferentiation via the Ca^2+^-calcineurin- (CaN-) NFAT pathway. (a) Quantification of CaN activity in cultured neonatal WT mouse primary CFs treated with TGF-*β* or transfected with negative control (NC) adenoviral vector (not shown), Ad-TRPA1 (Ad), negative control (NC) siRNA (not shown), or si-TRPA1 (si) and treated 48 hr later with TGF-*β*. *n* ≥ 3 per group. (b and c) Immunofluorescence staining (b) and quantification (c) of *α*-SMA-positive (red) stress fibers and DAPI (blue) in CFs treated with TGF-*β* or transfected with Ad-TRPA1 and stimulated 48 hr later with TGF-*β*, with and without FK506 treatment (scale bars = 25 *μ*m). *n* ≥ 3 per group. (d) *α*-SMA-luciferase promoter activity in CFs treated as described above. (e and f) Images (e) and quantification (f) of the contraction of floating collagen gel matrices seeded with CFs treated as described above. *n* ≥ 3 per group. (g and h) Western blot analysis and quantification of NFATc3 protein expression in the cytosolic and nuclear fractions of CFs treated as described above. Molecular weights in kDa are shown to the right of the blots. *n* ≥ 3 per group. The error bars represent the means ± s.e.m.^∗^ *P* < 0.05 and ^∗∗^ *P* < 0.01. The data are representative of the results of three or more independent experiments.

The biological effects of TRP channels (except for TRPM4 and TRPM5) are mediated mainly through Ca^2+^ signaling, in which the CaN-NFAT pathway has been particularly implicated [26]. The cellular CaN activity assay showed that the endogenous CaN activity was significantly enhanced by TRPA1 overexpression and attenuated by inhibition of TRPA1 expression (*P* < 0.05 and *P* < 0.05, respectively) (Figure 6(a)). CFs transfected with Ad-TRPA1 and treated with TGF-*β* showed *α*-SMA-positive stress fibers (*P* < 0.01), while treatment with the CaN inhibitor FK506 blocked this conversion and FK506 alone had negligible effects (Figures 6(b) and 6(d)). Moreover, FK506 abrogated collagen gel contraction in CFs stimulated with Ad-TRPA1 and TGF-*β* (*P* < 0.01) (Figures 6(e) and 6(f)). NFATc3 is normally localized in both the cytoplasm and nucleus of quiescent fibroblasts [27]. To further determine whether TRPA1 regulates NFATc3 expression and activity, we quantified nuclear NFATc3 localization by Western blotting in isolated CFs. Ad-TRPA1 and TGF-*β* treatment significantly reduced the NFATc3 protein level in the cytosolic fraction and increased it in the nuclear fraction (*P* < 0.01 and *P* < 0.01, respectively), whereas FK506 treatment decreased the protein level of nuclear NFATc3 (*P* < 0.05) (Figures 6(g) and 6(h)). Taken together, these results indicate that activation of the Ca^2+^-calcineurin-NFAT pathway is sufficient to drive TRPA1- and TGF-*β*-dependent CMF transdifferentiation.

### 3.7. DYRK1A-Regulated, CaN-Mediated NFATc3 Nuclear Localization Underlies CMF Transdifferentiation

**Figure 7:**
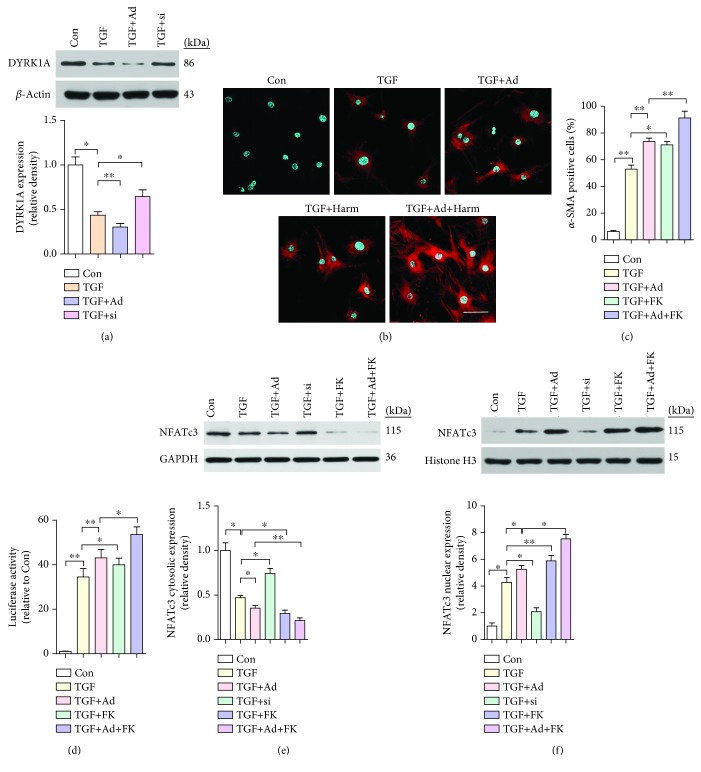
DYRK1A inhibits CaN-mediated NFATc3 nuclear localization in CMF transdifferentiation. (a) Western blot analysis and quantification of DYRK1A protein expression in neonatal WT mouse cultured primary CFs treated with TGF-*β* or transfected with negative control (NC) adenoviral vector (not shown), Ad-TRPA1 (Ad), negative control (NC) siRNA (not shown), or si-TRPA1 (si) and treated 48 hr later with TGF-*β*. Molecular weights in kDa are shown to the right of the blots. *n* ≥ 3 per group. (b and c) Immunofluorescence staining (b) and quantification (c) of *α*-SMA-positive (red) stress fibers and DAPI (blue) in CFs treated with TGF-*β* or transfected with Ad-TRPA1 and stimulated 48 hr later with TGF-*β*, with and without harmin (Harm) treatment. (d) *α*-SMA-luciferase promoter activity in CFs treated as described above (scale bars = 25 *μ*m). *n* ≥ 3 per group. (e and f) Western blot analysis and quantification of NFATc3 protein expression in the cytosolic and nuclear fractions of CFs treated as described above. Molecular weights in kDa are shown to the right of the blots. *n* ≥ 3 per group. The error bars represent the means ± s.e.m.^∗^ *P* < 0.05 and ^∗∗^ *P* < 0.01. The data are representative of the results of three or more independent experiments.

DYRK1A, which is mainly located in the nucleus, has been demonstrated to drive the nuclear export of NFAT [28]. We found that compared with treatment with TGF-*β* alone, transfection of CFs with Ad-TRPA1 reduced DYRK1A expression (*P* < 0.01) (Figure 7(a)). As stated in Section 3.6, more than 70% of CFs transfected with Ad-TRPA1 and treated with TGF-*β* showed *α*-SMA-positive stress fibers (*P* < 0.05), while treatment with the specific DYRK1A inhibitor harmine enhanced this conversion and harmine alone had no effect (Figures 7(b) and 7(d)). In addition, we examined the influence of DYRK1A on the intracellular localization of NFAT in CFs by Western blotting. Treatment with harmine resulted in increased nuclear translocation of NFATc3 after TRPA1 overexpression and TGF-*β* treatment (*P* < 0.05) (Figures 7(e) and 7(f)). In summary, transfection of CFs with Ad-TRPA1 reduced DYRK1A expression and promoted NFATc3 nuclear translocation and CMF differentiation.

## 4. Discussion

**Figure 8:**
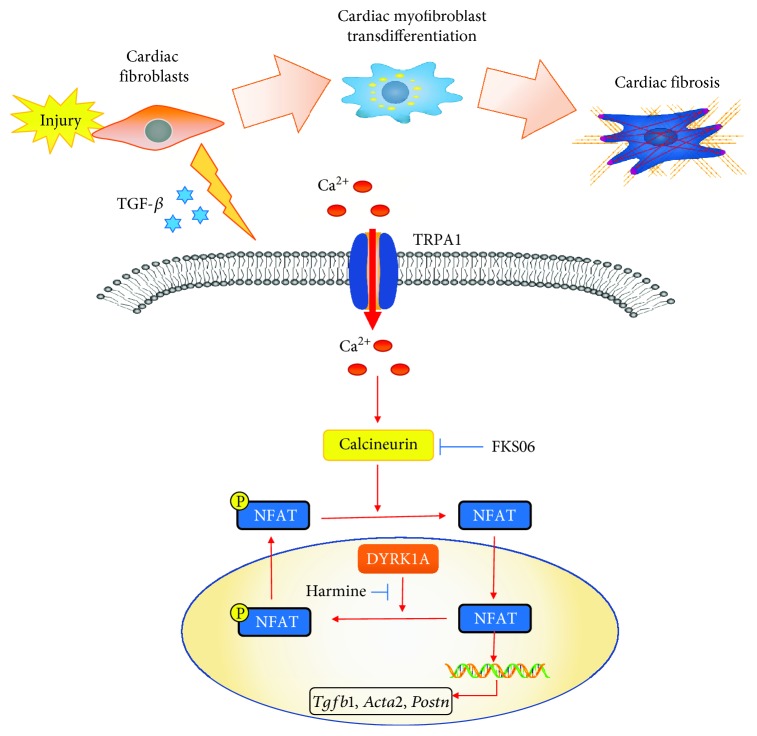
Schematic of the proposed mechanism underlying TRPA1-mediated CMF transdifferentiation and cardiac fibrosis after MI injury. Signaling model for CMF transdifferentiation whereby MI injury upregulates TRPA1 expression, enhancing Ca^2+^ entry and leading to CaN activation, resulting in the translocation of NFAT to the nucleus to participate in CMF phenotypic conversion. DYRK1A, as the downstream signaling effector of TRPA1, mediates NFAT transcription factors as likely regulators of CMF transdifferentiation-related gene expression.

The results of this study identified a new regulatory mechanism underlying CMF transdifferentiation, whereby TRPA1 is a mediator in the molecular pathways orchestrating CMF transdifferentiation and the cardiac fibrotic response after MI injury. TRPA1 expression, which is relatively low in uninjured heart tissue and quiescent CFs, is induced in CFs in response to signals from the MI-injured environment. With activation by agonists or upregulation of expression, TRPA1 then activates the TGF-*β*-Ca^2+^-CaN-NFAT signaling pathway, which mediates the transdifferentiation of *α*-SMA-positive CMFs and cardiac fibrosis. In addition, we discovered a previously unrecognized function of DYRK1A that is required for the function of the CaN-NFAT pathway in programming CMF transdifferentiation (Figure 8).

Previous studies have reported that TRPA1 is associated with fibrosis in various tissues, including cardiac tissue [29–31]. As mentioned before, increasing attention has been devoted to the role of CFs involved in cardiac remodeling [32]. CFs regulate the environment of cardiac myocytes through paracrine signaling by cytokines such as TGF-*β* and by direct communication via cell-cell interactions [33]. Acute MI causes rapid necrotic and apoptotic loss of cardiomyocytes within the ischemic region, and thereafter, the activity of cardiac CFs becomes critical in buttressing the ventricular wall as the fibrotic scar forms over several days [34]. Three to seven days after MI injury, CFs transdifferentiate into CMFs that secrete an abundance of extracellular matrix proteins and express *α*-SMA to structurally support the necrotic area; *α*-SMA expression is maintained as the collagen-containing extracellular matrix and scar fully mature [35]. The fibrotic healing response is initially protective [4]. Deletion of genes activating CMFs in the infarcted mouse heart resulted in greatly increased lethality; these genes are needed for extracellular matrix formation, and their deletion results in a heart significantly more susceptible to ventricular wall rupture in the first week after MI injury, indicating that CMF transformation is required for healing acute MI injury [25, 36, 37]. CMFs and their associated *α*-SMA microfilaments create a contractile scar tissue assembly [38]. Studies have demonstrated the contractile behavior of scar tissue. Scar formation and contraction contribute to the preservation of pump function at the early stage of postinfarction remodeling [39]. Additionally, CMFs in the infarct region replace lost cardiomyocytes and mediate the production of durable scar tissue, which helps to prevent infarct expansion and ventricular dilatation [40]. Therefore, CMF transformation is a critical player in the restoration of cardiac function, and early postinfarction remodeling could be beneficial and could promote survival [41] (Figure 1).

Previous studies provided evidence showing that TRPA1 expression was observed in human CFs, but the functional role of TRPA1 in CFs is still poorly understood [14]. In this study, we used pretreatment with the TRPA1 agonist cinnamaldehyde (CA) and TRPA1 knockout (KO) mice to gain the first understanding of the role of TRPA1 in cardiac fibrosis after MI injury. These results suggest that TRPA1 is necessary for MI injury-induced cardiac fibrosis (Figure 1 and Figure 2). Recently, there is a growing body of evidence implicating TRPA1 as a regulator of fibroblast-to-myofibroblast transdifferentiation [8, 42]. TGF-*β*, which activates myofibroblast transdifferentiation, is considered a fundamental initiator cytokine [32]. In our current study, KO MI mice had a significant reduction in the TGF-*β* level in the heart tissue lysate supernatant (Figure 1). Then, we conducted an *in vitro* experiment by culturing primary CFs in the presence of TGF-*β* to regulate the expression of TRPA1 (Figures 3 [Fig fig4] [Fig fig5] [Fig fig6]–7). These data indicate that TRPA1-mediated transdifferentiation is vital to CFs through inducing CMF formation.

Several lines of evidence have demonstrated that Ca^2+^ entry is essential for fibroblasts' biological functions and myofibroblast transformation [43, 44]. TRPA1 channels conduct mixed cation currents with a large Ca^2+^ fraction, and activation of TRPA1 on the plasma membrane of cells transiently creates small microdomains with highly localized Ca^2+^ concentrations [45]. Reports have shown that TRPA1-mediated Ca^2+^ influx is stimulated by the direct application of the selective agonists CA or AITC [46]. Elementary Ca^2+^ signals arising from the influx of Ca^2+^ through single TRPA1 channels were recorded and called “TRPA1 sparklets” [47]. In this study, Ca^2+^ influx and intracellular calcium were optically investigated by loading with the Ca^2+^ indicator dye Fluo-4 and imaging using confocal microscopy, and it is possible that TRPA1 mediates Ca^2+^ entry into CFs to intensify the fibrotic phenotype (Figure 5).

Calcium influx into CFs through different TRP family channels is critical for maintaining CMF transdifferentiation by regulating CaN-NFAT-dependent target genes implicated in cardiac fibrosis [27, 48]. A conformational change exposes the active site on CaN, leading to NFAT dephosphorylation and translocation to the nucleus, where it triggers the transcription of genes such as *Col3* and *Acta2* [27]. These results actually support our current results: TRPA1 upregulation increased the cytosolic calcium concentration ([Ca^2+^]c), which enhanced CaN activity and NFATc3 nuclear translocation in CFs, thereby also promoting CMF transdifferentiation; however, this activity was blocked by the CaN inhibitor FK506 (Figure 6).

DYRK1A belongs to a family of dual-specificity kinases and is ubiquitously expressed at high levels in the developing nervous system and the heart [49]. DYRK1A, which is mainly localized in the nucleus, has been identified as a novel modifier of NFAT transcription factors. DYRK1A directly phosphorylates NFATs and drives their nuclear export [23]. Our data imply that TRPA1 and DYRK1A are negatively correlated in CFs treated with TGF-*β*. Moreover, inhibition of DYRK1A and constitutively active CaN led to robust induction of NFAT nuclear translocation, leading to CMF transdifferentiation at the morphological and molecular levels (Figure 7). Although these changes have been shown to occur in cardiomyocytes involved in myocardial hypertrophy [50], the present research constitutes the first demonstration of TRPA1-associated DYRK1A changes in CFs and the involvement of these CFs in MI-related CMF transdifferentiation.

Although TRPA1 is a clearly attractive agent to consider for the treatment of cardiac fibrotic disease states, several limitations remain. First, we need to observe the effect of TRPA1 in different stages of cardiac fibrosis caused by MI and to consider the timing of antifibrotic therapies so that the MI injury healing phase can be effectively maintained. Our results showed that in the primary stage of postinfarction remodeling, *Trpa1^−/−^* mice had greater reductions in cardiac function and observably greater ventricular wall dilation than *Trpa1^+/+^* mice (Figures 1(b) and 1(c)). Second, in this study, the mechanisms of TRPA1 as an anti-CMF transdifferentiation mediator were explored *in vitro* but were less thoroughly confirmed *in vivo*. Because *in vitro* cell experiments cannot fully simulate *in vivo* conditions, the weak link between *in vivo* and *in vitro* studies should be taken into consideration. Therefore, determining further approaches to optimize the potency of TRPA1 functions *in vivo*, unequivocally defining the downstream molecular targets of TRPA1, and developing methods to specifically direct TRPA1 to cardiac fibrosis are key future challenges.

## 5. Conclusion

Taken together, the data presented here demonstrate that TRPA1-Ca^2+^ influx-CaN-NFAT signaling is an integral mechanism underlying the rapid response of CFs to MI injury via CMF transdifferentiation. More importantly, we observed the mechanism by which TRPA1 enhances CaN-NFAT-mediated cardiac fibrosis by blocking DYRK1A. The identification of this signal transduction mechanism within CFs helps to predict additional keys to developing a targeted intervention approach for cardiac fibrosis.

## Data Availability

The data used to support the findings of this study are available from the corresponding author upon request.

## Supplementary Materials

Supplementary Materials Supplementary Figure S1: TRPA1 expression in primary neonatal wild-type (WT) mice cardiac fibroblasts (CFs) transfected with Ad-TRPA1 or si-TRPA1. (A) Western blot analysis and quantification of the TRPA1 protein level in primary neonatal WT mice CFs, with and without transfecting Ad-TRPA1. Molecular weights in kDa are shown to the left of the blots. *n* ≥ 3 per group. (B) Western blot analysis and quantification of the TRPA1 protein level in primary neonatal WT mice CFs, with and without transfecting si-TRPA1. Molecular weights in kDa are shown to the left of the blots. *n* ≥ 3 per group. Error bars represent the means ± s.e.m.^∗^ *P* < 0.05 and ^∗∗^ *P* < 0.01. The data are representative of three or more independent experiments.
