# Supplementary material for: TRPA1 Promotes Cardiac Myofibroblast Transdifferentiation after Myocardial Infarction Injury via the Calcineurin-NFAT-DYRK1A Signaling Pathway
Source: Oxid Med Cell Longev. 2019 May 14;2019:6408352. doi: 10.1155/2019/6408352 (PMC6537015; doi:10.1155/2019/6408352)
Supplement: Supplementary Materials — Supplementary Figure S1: TRPA1 expression in primary neonatal wild-type (WT) mice cardiac fibroblasts (CFs) transfected with Ad-TRPA1 or si-TRPA1. (A) Western blot analysis and quantification of the TRPA1 protein level in primary neonatal WT mice CFs, with and without transfecting Ad-TRPA1. Molecular weights in kDa are shown to the left of the blots. n ≥ 3 per group. (B) Western blot analysis and quantification of the TRPA1 protein level in primary neonatal WT mice CFs, with and without transfecting si-TRPA1. Molecular weights in kDa are shown to the left of the blots. n ≥ 3 per group. Error bars represent the means ± s.e.m.∗ P < 0.05 and ∗∗ P < 0.01. The data are representative of three or more independent experiments. [file 6408352.f1.docx]

Supplementary Materials

Figure S1


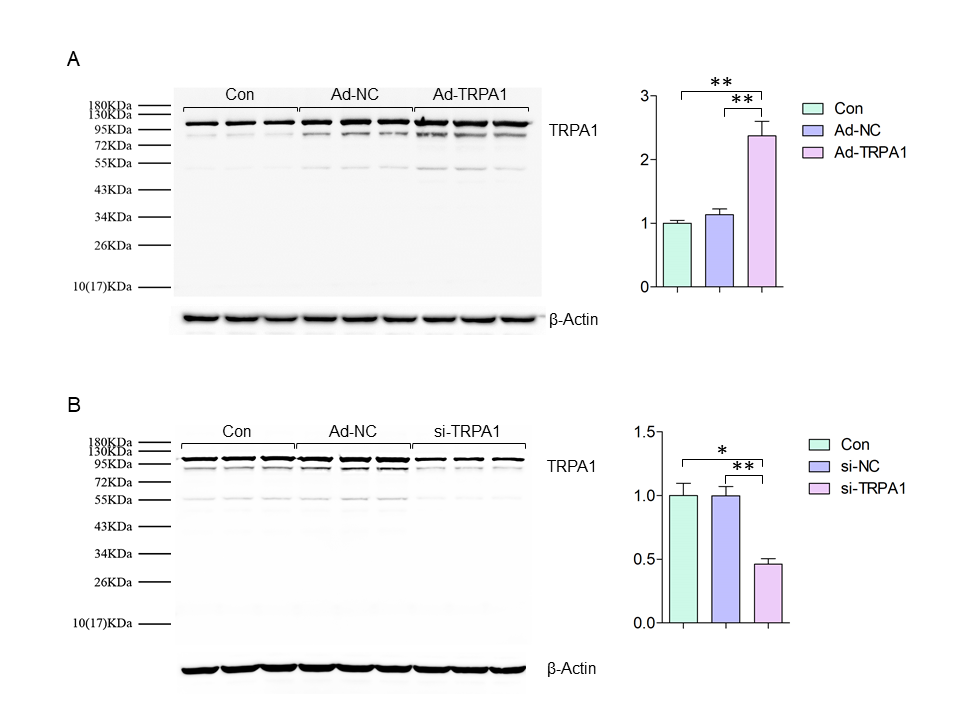


Figure S1：TRPA1 expression in primary neonatal wild type (WT) mice cardiac fibroblasts (CFs) transfected with Ad-TRPA1 or si-TRPA1.

A. Western blot analysis and quantification of the TRPA1 protein level in primary neonatal WT mice CFs, with and without transfecting Ad-TRPA1. Molecular weights in kDa are shown to the left of the blots. n≥3 per group.

B. Western blot analysis and quantification of the TRPA1 protein level in primary neonatal WT mice CFs, with and without transfecting si-TRPA1. Molecular weights in kDa are shown to the left of the blots. n≥3 per group.

Error bars represent the means ± s.e.m. * *p* < 0.05 and ***p* < 0.01. The data are representative of three or more independent experiments.
